# Catalytic Adsorptive Stripping Voltammetric Determination of Germanium Employing the Oxidizing Properties of V(IV)-HEDTA Complex and Bismuth-Modified Carbon-Based Electrodes

**DOI:** 10.3390/membranes11070524

**Published:** 2021-07-13

**Authors:** Agnieszka Królicka, Jerzy Zarębski, Andrzej Bobrowski

**Affiliations:** Department of Building Materials Technology, Faculty of Materials Science and Ceramics, AGH University of Science and Technology, Mickiewicza 30, 30-059 Krakow, Poland; abobrow@agh.edu.pl (J.Z.); zarebski.jerzy@gmail.com (A.B.)

**Keywords:** germanium determination, vanadium(IV)-HEDTA complex, catalytic adsorptive stripping voltammetry (CAdSV), bismuth film, glassy carbon, screen-printed electrodes

## Abstract

An efficient procedure that may be used to determine germanium traces and combines the advantages of catalytic adsorptive stripping voltammetry (CAdSV) with the convenience of screen-printed electrodes was developed. To induce the CAdSV response of the germanium(IV)-catechol complex, the vanadium(IV)-HEDTA compound was employed in combination with various bismuth-modified homogeneous (glassy carbon, gold coated with a bismuth layer via physical vapor deposition) and heterogeneous (screen-printed carbon, mesoporous carbon, graphene and reduced graphene oxide, polymer-encapsuled carbon fiber) electrodes. This solution had never before been implemented for this purpose. To achieve the most favorable performance of the working electrode, the parameters of bismuth deposition were optimized using a central composite design methodology. SEM imaging and contact angle measurements confirmed the long-term stability and high chemical resistance of the electrodes against the oxidizing action of V(IV)-HEDTA. Under optimized conditions, the method made it possible to detect nanomolar concentrations of germanium with favorable detection limits, high sensitivity, and a wide linear range of 5–90 nM of Ge(IV).

## 1. Introduction

Germanium, an element that exhibits the characteristics of both metals and non-metals, represents a unique group of materials known as Critical Raw Materials (EU) [1] or Critical Minerals (USA) [2]. Although such elements typically constitute only a small percentage of a material or product by weight, they give it key chemical or physical properties, and are thus essential to its performance [3]. The common features of critical materials are their limited availability and rapidly growing demand from manufacturers of modern devices. In the EU, germanium is used mainly in the production of optical fibers, infrared optics, and solar cells for satellite applications. This is because Ge-based photovoltaic cells offer much higher efficiency than their silicon-based counterparts. As is the case with many critical materials, germanium does not occur naturally in its elemental state and is rarely the main component in minerals (germanite, a rare mineral from the sulfide group, being the exception). Germanium is produced on an industrial scale mostly using either sphalerite—a zinc sulfide mineral—or fly ash, but improving yield remains a major challenge. To meet the demands of modern technologies, it is necessary to either increase germanium supply by finding new deposits of Ge-containing minerals, improve the efficiency of mineral processing, or—what seems to be the most feasible solution—promote its recovery from electronical devices (IR cameras) or optical fibers [4].

Bearing in mind that the Ge content in geological materials and waste is generally very low and rarely exceeds the mg/kg level, analytical procedures used to determine germanium must be very sensitive. The techniques most frequently applied for this purpose are different spectrometric methods featuring inductively coupled plasma, i.e., inductively coupled plasma optical emission spectrometry (ICP-OES) or inductively coupled plasma mass spectrometry (ICP-MS). Metal determination with ICP-MS, despite the very high sensitivity of this technique, may be difficult due to the dissolved salts found in the sample as well as spectral interference. It is therefore necessary to use additional procedures aimed at removing these salts and/or concentrating the sample via precipitation on a support, the use of ion exchange resins, or special sprays. However, voltammetric techniques are insensitive to the presence of inorganic salts and at the same time offer low detection limits. Among voltammetric methods, catalytic adsorptive stripping voltammetry (CAdSV) plays a uniquely important role in trace analysis due to its remarkable sensitivity [5,6,7,8,9]. The CAdSV procedures that may be used for germanium determination were reviewed in our previous papers [10,11,12]. In general, the complexes that Ge(IV) forms with organic ligands in the examined solution are adsorbed on the surface of the working electrode and then they induce the catalytic reduction of certain oxidants, such as BrO_3_^−^ [13,14], V(IV) [15], V(IV)-EDTA [16,17,18] or, most recently, V(IV)-HEDTA (HEDTA: N-(2-hydroxyethyl)-ethylene diamine N,N′,N′-triacetic acid) [10,11,12]. The derivatives of tri- or tetraacetic acids (HEDTA, EDTA, NTA—nitrilotriacetic acid) are not only applied in CAdSV but are also used as reagents capable to functionalize the surface of nanomaterials such as nanoparticles [19] or carbon nanotubes [20,21]. The catalytic system that utilizes V(IV)-EDTA as the oxidant offers extremely high sensitivity and low LOD (0.01 nM of Ge(IV) [10] when the mercury electrode is used. To meet the current guidelines that impose limitations on the application of mercury, environmentally friendly electrodes should be employed instead. Bismuth film electrodes (BiFEs) in particular have been popular as they offer many of the advantages of mercury electrodes (wide range of accessible potential, chemical inertness, high sensitivity towards many inorganic and organic analytes), and at the same time are devoid of its disadvantages [22]. BiFEs plated in situ on glassy carbon disc-shaped supports were recently applied to determine germanium as part of an adsorptive stripping voltammetric procedure [23]. It seems that screen-printed electrodes (SPEs) can significantly contribute to the further development of relevant analytical procedures [24,25,26,27] since they can be mass-produced, disposable, inexpensive as well as ready-to-use, and can be used with portable electrochemical analyzers. The composition of inks can be easily modified with many materials, for example, different carbon allotropes [28]. Unfortunately, the binding polymers employed for the fabrication of inks for SPEs do not exhibit insufficient chemical resistance to aggressive oxidizing reagents. This factor strongly limits the applicability of SPEs in CAdSV systems [29].

## 2. Materials and Methods

### 2.1. Instrumentation

Electrochemical study was performed on a Autolab 204 analyzer (Metrohm Autolab, Herisau, Switzerland). Disposable screen-printed electrodes (4 mm diameter) with ceramic backing (DropSens, Oviedo, Spain), disc electrodes (2 and 3 mm diameter) made of glassy carbon, gold, and platinum (Mineral, Poland), carbon multi fiber (5 µm in diameter, lab made, Scheme S1, Supplementary Material) electrodes were used as supports for bismuth films. Platinum wire and Ag/AgCl(3M KCl) were applied as the anode and reference electrodes. To record the voltametric curves, the DP mode was used with a pulse amplitude of 50 mV. Solutions were stirred during the deposition step, which was followed by 5 s of equilibration.

Static contact angles of the bismuth films were measured by Attention Theta tentiometer (Biolin Scientific, Espoo, Finland).

### 2.2. Reagents

Unless otherwise specified, reagents were used as received without further purification. All solutions were prepared using deionized water with a resistivity of 18.2 MΩ (Millipore, Simplicity UV). A 0.2 M VOSO_4_ solution was prepared by dissolving 0.9094 g of V_2_O_5_ (POCH, Gliwice, Poland) in a solution containing 1 mL of 96% H_2_SO_4_ (Suprapur, Merck, Darmstadt, Germany) and 1.5 g of oxalic acid (POCH, Gliwice, Poland), heated in a water bath. After the complete dissolution of V_2_O_5_, the solution was evaporated until the appearance of sulfuric acid fumes to decompose the excess of oxalic acid. After cooling, the evaporated solution was transferred to a 50 mL volumetric flask, which was then filled to a volume with water. An acetate buffer was prepared by adding 30% NaOH (Suprapur, Merck, Germany) to a diluted solution of 96% acetic acid (POCH, Poland) and water—up to the required pH while mixing, using a pH meter. Solution of the complexes of vanadium with HEDTA (min. 99%, HEDTA, Fluka, Germany) were prepared by mixing appropriate amounts of a 0.2 M solution of VOSO_4_ with solution HEDTA and fixing their pH to the pH value of the applied acetate buffer. Catechol solutions were prepared daily and was kept in the refrigerator. Bismuth films were prepared by electrolysis of bismuth(III) solution in 0.34 M HClO_4_ at ambient temperature. Caution: hot concentrated solutions of perchloric acid can be extremely dangerous (explosion hazard and fire hazard).

### 2.3. Ex-Situ Electrode Preparation

Bismuth films were plated just prior to use by means of potentiostatic deposition. Before plating, the disc substrates were polished using an Al_2_O_3_ suspension (0.3 and 0.05 µm) applied onto a polishing cloth. Screen-printed electrodes did not require any preparation or processing other than 2 min of soaking in the plating solution immediately prior to electrolysis. The plating process was monitored by recording chronoamperometric curves and stopped when the charge reached the defined threshold. Pre-plated electrodes were rinsed with 0.34 M HClO_4_ and water.

### 2.4. Catalytic Adsorptive Stripping Determination of Ge(IV)

The developed analytical procedure required the use of a supporting electrolyte comprising 0.05 M acetate buffer (pH of 4.4), 1 mM of catechol, 1 mM of V(IV), and 1.5 mM of HEDTA, which made it possible to induce the catalytic action of the Ge(IV)-catechol-V(IV)-HEDTA system [10]. CAdSV voltammograms were recorded after 30 s of accumulation performed at the potential of −0.4 V—parameters obtained as a result of optimization studies.

### 2.5. Design of Experiments

A central composite design (CCD) was applied to study the effect of plating potential (E_plat_, designated by x_1_), the charge transferred during electrolysis (Q—x_2_), and the concentration of bismuth ions in the plating solution (c—x_3_) on the germanium peak current and its geometry. GC discs with a diameter of 3 mm were used as supports. The full quadratic model for three factors with three levels was employed. Table S1 (Supplementary Material) shows the design matrix in which the variables (E_plat_, Q, c), the coded levels used, the decoded variables, and the values of germanium peak current and the half-width peak potential are provided. Each of the 15 combinations ran in a random order in two trials (t_1_ and t_2_). Microsoft Excel was used for calculations and as a random number generator. Surface plots were constructed using the OriginPro 2021 software.

### 2.6. Contact Angle Measuring

The bismuth films were deposited on four glassy carbon discs (8 mm in diameter) via the electrolysis of a 0.04 M Bi(III) solution in 0.34 M HClO_4_ at −0.9 V. To avoid contamination with the products of Bi(III) ion hydrolysis, the surface of the bismuth films was cleaned using 0.34 M HClO_4_, then rinsed with deionized water and air-dried (T = 20 ± 3 °C, relative humidity = 35 ± 5%). After the cleaning procedure, a 4 µL droplet of deionized water was deposited by means of the sessile drop technique onto the bismuth film. Static contact angles of the as-prepared coatings were measured with the Attention Theta Lite tensiometer (Biolin Scientific, Finland).

## 3. Results and Discussion

### 3.1. Selection of an Optimal Support for Bismuth Film Deposition

**Figure 1:**
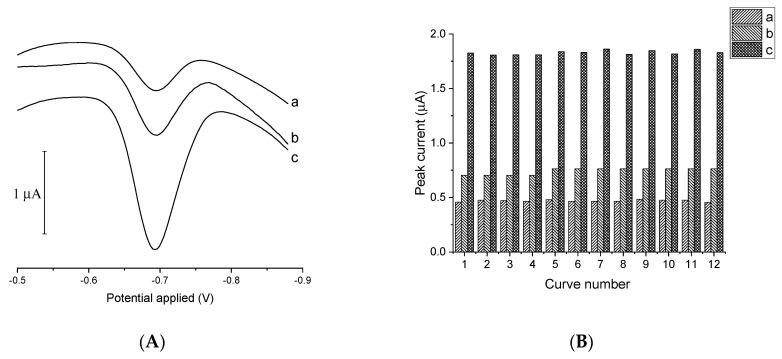
(**A**) Voltammograms recorded in a solution containing 25 nM of Ge(IV) using bismuth films plated on Au (a), Pt (b), and GC (c) disc electrodes. (**B**) Ge(IV) peak currents determined from twelve successive voltametric measurements involving Au (a), Pt (b), and GC (c) disc electrodes plated with Bi film. Composition of the supporting electrolyte: 0.05 M acetate buffer, 1 mM of catechol, 1 mM of V(IV), and 1.5 mM of HEDTA. Instrumental parameters: accumulation time 30 s, accumulation potential −0.4 V. Electrode preparation: electrodeposition at −0.9 V in a 0.04 M Bi(III) solution, carried out for a period sufficient to transfer a charge of 7.07 mC per mm^2^ of the surface area of the support electrode.

Catalytic adsorptive stripping voltametric procedures used for the determination of metal cations are very sensitive, but to achieve effective signal amplification it is necessary to use strongly oxidized reagents. These aggressive reagents can damage the metal and organic layers used as working electrodes. Strong oxidizers such as nitrite, nitrate, bromate, and chlorate prevent screen-printed electrodes from being used as working electrodes in catalytic stripping voltammetry. This is because of the insufficient resistance of the binders to the action of these chemicals, which often causes the cohesion and adhesion of the printed layers to become progressively worse. To test whether the V(IV)-HEDTA complex recently introduced as a catalytic agent for extremely sensitive germanium quantification [10] might be applied together with pre-plated BiFE, different variants of bismuth-plated electrodes were investigated. First, glassy carbon, gold, and platinum disc electrodes were plated with bismuth and tested as potential supports. The recorded CAdSV signals of germanium obtained using Bi/Au, Bi/Pt, and Bi/GC electrodes by means of a recently elaborated procedure [10] were well-developed and highly reproducible (RSD < 4%), as shown in Figure 1. The bismuth layer seemed to be stable and retained its electrochemical activity when exposed to the solution containing the V(IV)-HEDTA complex. In the next step, heterogeneous supports comprising carbon particles or carbon fibers dispersed in the binding polymers, i.e., various SPEs, were tested. All tested composite materials proved to be stable supports for bismuth films, maintaining structural integrity when exposed to the V(IV)-HEDTA solution.

### 3.2. Optimization of Bismuth Plating with the Use of Central Composite Design

Following the initial BiFE evaluation, the ability of bismuth layers to provide sensitive CAdSV germanium signals was investigated further. The influence of three factors affecting the bismuth film morphology and therefore the properties of the electrodes—the plating potential, plating charge, and the concentration of the bismuth plating solution—on the electrochemical performance of the bismuth film electrodes plated on glassy carbon was examined. The boundary values were selected to provide the conditions necessary for the formation of dendrite-like bismuth structures, known to yield desirable performance in voltametric applications [30,31]. To study the simultaneous action of the three plating variables on the performance of the BiFEs, the central composite design was employed. A design matrix for the investigated factors, which contains data entries in coded units (x_1_–x_3_), their real values, and the obtained results, is presented in Table S1 (Supplementary material).

**Figure 2:**
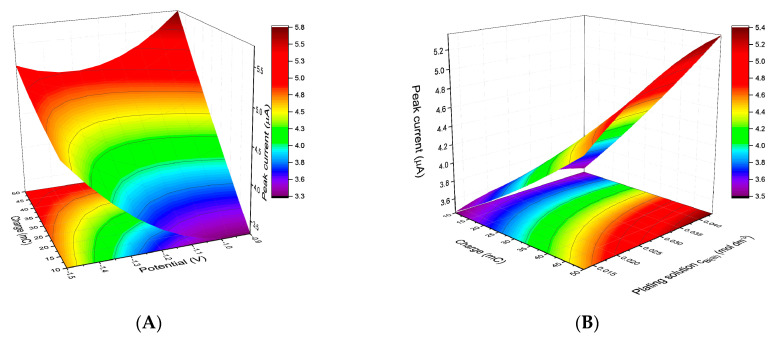

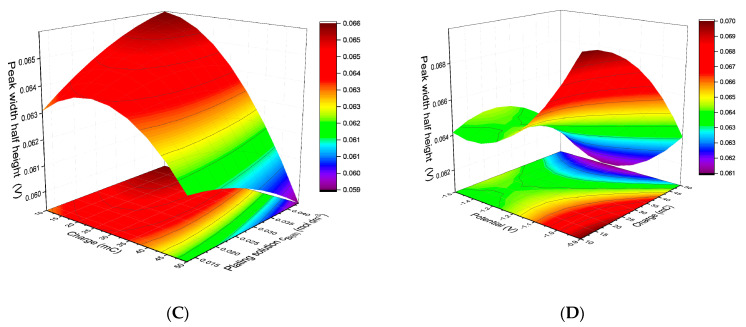
Response surfaces for Ge(IV) peak current (**A**,**B**) and the half width potential of Ge(IV) (**C**,**D**).

The relationship between the variables listed above and the geometry of the germanium peak (peak current, Ip, and half-width peak potential, w_1/2_) was studied using contour and response surface plots. As shown in Figure 2A,B, the highest germanium signal was achieved when the investigated value of the plating potential was −0.9 V and the other variables had a maximum value (50 mC for an electrode with an area of 7.07 mm^2^ and a Bi(III) ion concentration of 0.04 M). The narrowest germanium peaks were observed when the largest charge and the highest concentration of the plating solution were applied simultaneously (Figure 2C). When testing the influence of plating potential on the half-width peak potential, a more complex interdependence was revealed (Figure 2D). When analyzing the curvature of the surface plot, two main trends can be distinguished: (1) for a given potential value, the half-width peak potential decreased with increasing plating charge and (2) for a given charge, the minimum of the w_1/2_ = f (E_plat_) curve was within the range of −1.2 to −1.4 V. For 50 mC, i.e., the value that yielded the highest peak current, the difference between the most favorable and least favorable response was only 0.003 V and the impact of plating potential on w_1/2_ can be considered as rather limited. Since the bismuth films obtained in the 0.04 M Bi(III) solution after electrolysis at an applied charge of 50 mC and a voltage of −0.9 V were determined to perform the best, they were selected for the subsequent, more comprehensive study.

### 3.3. Surface Morphology

**Figure 3:**
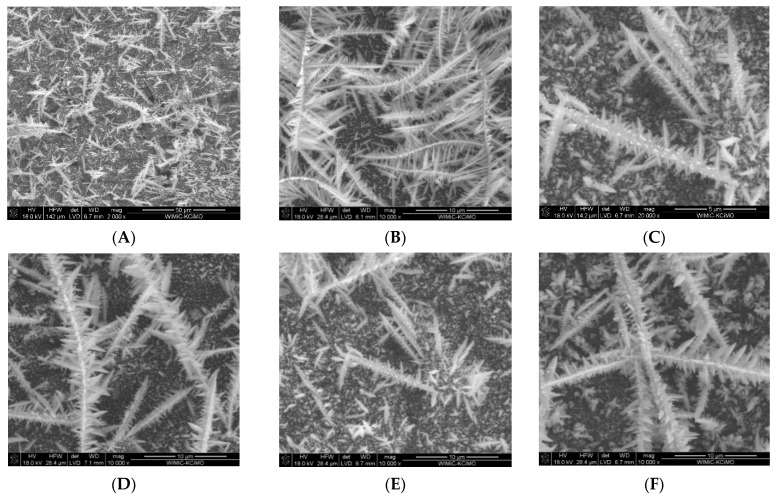
SEM images of bismuth layers deposited on GC (**A**–**C**) and screen-printed supports: carbon (**D**), mesoporous carbon (**E**), and ordered mesoporous carbon (**F**). Plating parameters: solution 43 mM Bi(III) in 0.34 M HClO_4_, plating potential −0.9 V, plating charge 7 mC per mm^2^ of the support surface.

The bismuth film surface was first characterized by means of scanning electron microscopy. Figure 3 shows the SEM images of the bismuth film deposited onto a glassy carbon disc (Figure 3A–C) and three carbon-based screen-printed electrodes—carbon (Figure 3D), mesoporous carbon (Figure 3E), and ordered mesoporous carbon (Figure 3F).

As Figure 3 demonstrates, every tested carbon support was evenly covered by a layer of three-dimensional dendritic structures that resembled fronds. Under a magnification of 20,000, it became apparent (Figure 3C) that every branch comprised abundant tiny subbranches which had a diameter of 0.3 µm. In addition, the surface of the support was dotted with numerous crystalline objects, including small (less than 0.1 µm in diameter) cones. EDS analysis of the bismuth layers on different supports indicated that the bismuth coverage was very high (from 68.8% for ordered mesoporous carbon to 92.5% for glassy carbon). The small oxygen content (1.3–3.5%) confirmed that the bismuth film was free from oxides or the products of bismuth ion hydrolysis, such as compounds containing BiO^+^.

### 3.4. Electrode Stability Test by Contact Angle Measurement

Since the surface microstructure of materials correlates closely with the apparent contact angle at the boundary between the liquid and the surface, wettability studies were conducted. The possible chemical (e.g., film oxidation, build-up of electroreduction products) and physical (e.g., exfoliation of deposited coatings, adsorption, water absorption) changes in properties of an electrode may be reflected in the contact angle.

**Figure 4:**
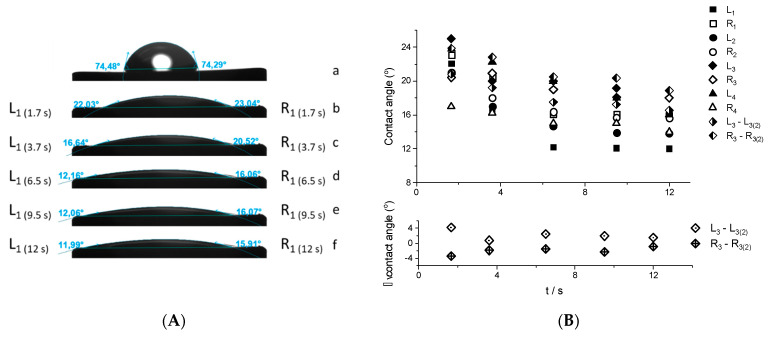
(**A**) Photographs illustrating the contact angle for water drops deposited onto a GC disc (a) and bismuth film plated on GC (electrode 1) (b−f). The photographs (b–f) show how the drop changed over the 12 s after deposition. (**B**) Values of left (L_1_, L_2_, L_3_, L_4_, and L_3(2)_ − second run for electrode 3) and right (R_1_, R_2_, R_3_, R_4_, and R_3(2)_ − second run for electrode 3) contact angles observed for four different BiF/GC electrodes (upper panel). Differences between the left (L_3_−L_3(2)_) and right (R_3_—R_3(2)_) contact angles values observed in the first and second run for electrode 3 (lower panel).

Bare and bismuth-plated carbon supports were cleaned with 0.34 M HClO_4_ and deionized water and then air-dried. A droplet of water was then deposited onto the examined surface by means of the sessile drop technique and a high-resolution camera captured its image for 12 s (Figure 4). The performed studies showed the bismuth film surface-plated on GC to be highly hydrophilic (average contact angle of 20 ± 2° measured 1.66 s after drop deposition) and homogeneous, as evidenced by the small difference between the left and right contact angles—3 ± 1° on average (Figure 4B). The surface of bare GC was also hydrophilic, but the contact angle was much higher—75 ± 6° on average. The wettability of the films did not change significantly during the dry-wet tests. In the case of the data shown in Figure 4A, the contact angles obtained for the drop placed on fresh bismuth film and that placed on the film previously exposed to water vary by ±2° on average (Figure 4B, lower panel). The low contact angle confirms that the three-dimensional dendritic bismuth layer structure is stable and resistant to mechanical damage. It also indicates that considerable roughness does not prevent the access of water molecules to the electrode surface, which tends to occur in superhydrophobic materials in which a rough surface can trap air, causing an increase in the water contact angle. The studies involving bismuth layers plated onto carbon SPEs lead to similar conclusions. The bare SPE supports were highly hydrophobic, but their hydrophobicity changed with time of contact with water. In the case of carbon SPEs, the contact angle, initially equal to 120°, steadily decreased to 105° when in contact with water. When plated with bismuth, the contact angle decreased to 40 ± 3°, regardless of the type of SPE support. In contrast to bare SPEs, no marked changes were observed after exposure to distilled water. To assess the durability of BiFEs in real-life electroanalytical conditions, the bismuth films were repeatedly exposed to the supporting electrolyte containing the V(IV)-HEDTA complex, catechol and acetate buffer, cleaned with water, and then air-dried. The contact angle was then measured using water and supporting electrolyte as the probe liquid and these tests revealed that the contact angle did not vary substantially over the duration of the analysis. These observations confirmed that the bismuth layers plated on both homogeneous and multicomponent carbon-based supports according to the optimized procedure may be useful as sensing layers in catalytic adsorptive stripping voltametric procedures utilizing the V(IV)-HEDTA complex as a catalytic agent. Consequently, the measurement of the contact angle seems to be a simple and effective evaluation tool allowing the usefulness of a film electrode in electroanalysis to be verified.

### 3.5. Analytical Performance

**Figure 5:**
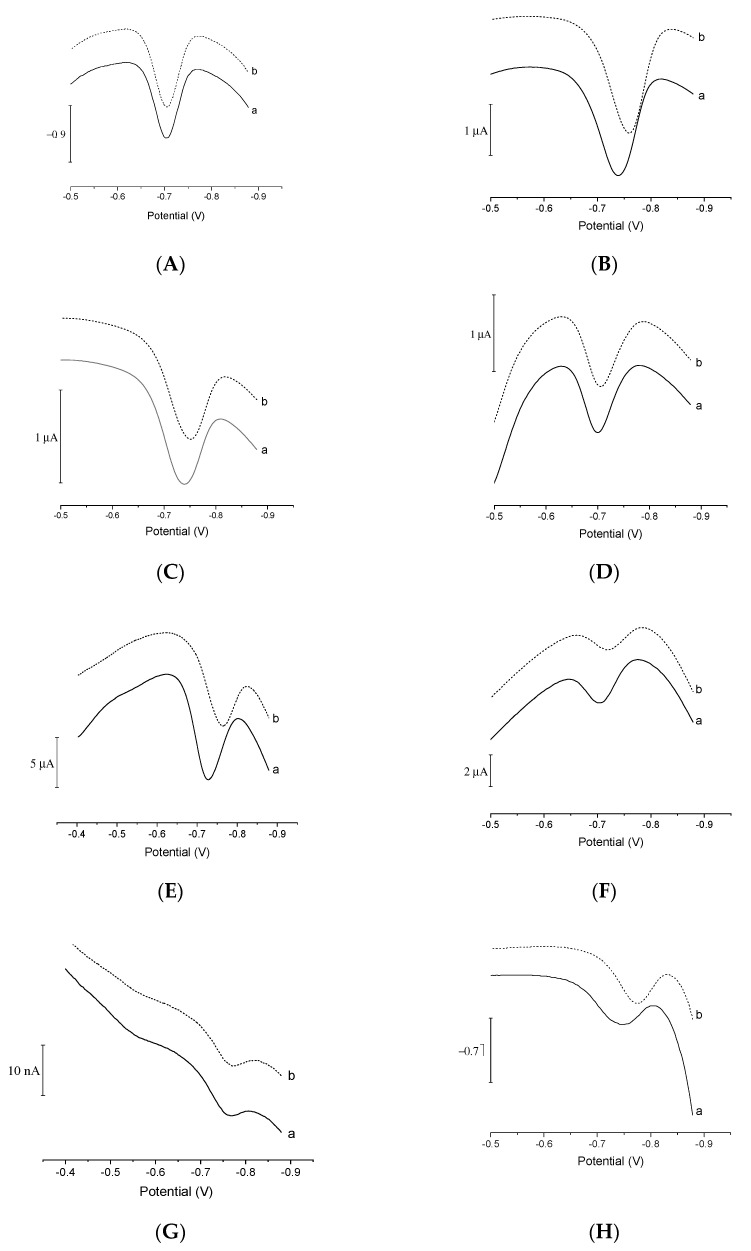
The first (a) and tenth (b) voltammogram recorded in a solution containing 30 nM of Ge(IV) (**A**−**F**) or 300 nM of Ge(IV) (**G**,**H**) using bismuth films plated on GC (**A**), carbon SPE (**B**), mesoporous carbon SPE (**C**), ordered mesoporous carbon SPE (**D**), graphene SPE (**E**), reduced graphene oxide SPE (**F**), carbon multifiber electrode (**G**), and bismuth-sputtered electrode (**H**). Other parameters as in Figure 1.

To make the best of the potential of the proposed CAdSV procedure of Ge(IV) determination and the advantages of bismuth film electrodes, eight types of electrodes were evaluated: (1) BiFE plated on GC (BiFE/GC), (2) BiFE plated on carbon screen-printed electrodes (BiFE/SPE), (3) BiFE plated on mesoporous carbon screen-printed electrodes (BiFE/SPE_meso_), (4) BiFE plated on ordered mesoporous carbon screen-printed electrodes (BiFE/SPE_or-meso_), (5) BiFE plated on graphene screen-printed electrodes (BiFE/SPE_g_), (6) BiFE plated on reduced graphene oxide screen-printed electrodes (BiFE/SPE_rGO_), (7) BiFE plated on carbon multifiber electrode (BiFE/F), and (8) bismuth-sputtered electrode (Bi_sp_). The voltammograms recorded using the electrodes listed above are shown in Figure 5. Each of the electrodes provided measurable germanium signals, but those obtained by means of BiFE/GC and carbon or mesoporous carbon were the most favorable, since they offered the most sensitive and reproducible voltammetric response—the relative standard deviation values of Ge(IV) peak currents for 30 nM of Ge(IV) were as follows: BiFE/GC = 4.5%, BiFE/SPE = 2.8%, BiFE/SPE_meso_ = 2.5%, BiFE/SPE_or-meso_ = 2.1%. The remaining electrodes were characterized either by signals that were asymmetrical (Bi_sp_) and/or non-reproducible (BiFE/SPE_rGO_, Bi_sp_), or a low signal-to-noise ratio (BiFE/F). In the case of BiFE/SPE_g_, BiFE/SPE_rGO_, Bi_sp_, and BiFE/SPE, the comparison of the first voltammogram (Figure 5, curves labeled a) and the tenth consecutive one (Figure 5, curves labeled b) recorded by means of the same electrode revealed a tendency of the germanium peak to shift to a more negative potential (Table S2). This suggests that the properties of the electrodes listed above undergo certain changes that could adversely affect their practical application. The more homogenous supports (e.g., GC or carbon fiber) offer a more stable potential of Ge(IV) peaks, as evidenced by comparison of the peak potentials observed on the first (E_p(a)_) and tenth (E_p(b)_) votlammograms. This is particularly apparent when Ge(IV) peaks were recorded by SPE electrodes constructed using carbon materials, characterized by a different extent of the long-range order, namely, carbon, mesoporous carbon, and ordered mesoporous carbon. The E_p(a)_–E_p(b)_ value was the smallest for BiFE/SPE_or-meso_ and the highest for BiFE/SPE, with BiFE/SPE_meso_ being in the middle. At the same time, the sensitivity of Ge(IV) signal decreased in the same order. As far as the selection of the optimal working electrode is concerned, a compromise solution should be found.

**Figure 6:**
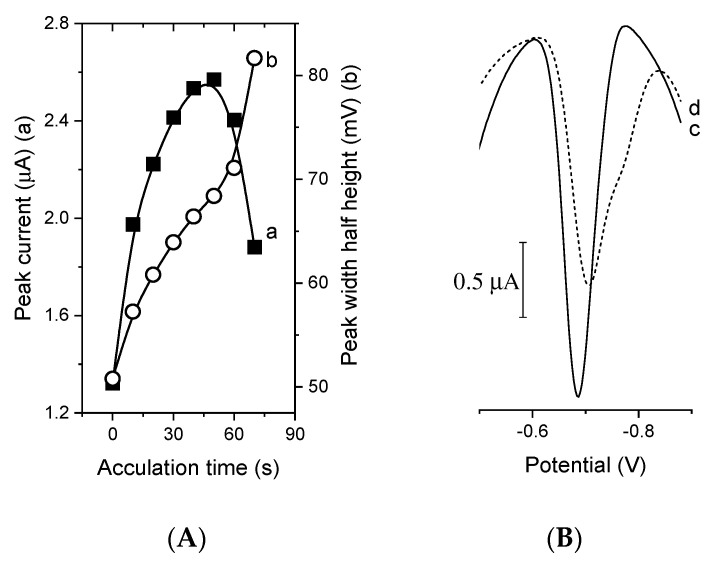
(**A**) Dependence of CAdSV peak current of Ge(IV) (a) and half width at half maximum (b) obtained using a BiF/GC electrode on accumulation time. (**B**) Voltammograms recorded after 30 s (c) and 70 s (d) of accumulation at a potential of −0.4 V. Other parameters as in Figure 1.

The ability of BiFEs to accumulate the germanium(IV) catecholate complex in the presence of V(IV)-HEDTA was examined by changing the deposition potential and time. The highest value of the catalytic peak current of Ge(IV) was achieved at −0.4 V. The dependence of the CAdSV peak current of Ge(IV) vs. accumulation time in the time range from 0 to 50 s (Figure 6A) was typical of processes with an adsorptive contribution, in which the current increases initially and then levels off due to the saturation of the surface of the electrode by the adsorbed complex. For longer accumulation times, the germanium peak widened unfavorably (Figure 6A, curve b) and a deformation in the form of a shoulder was observed (Figure 6B).

**Figure 7:**
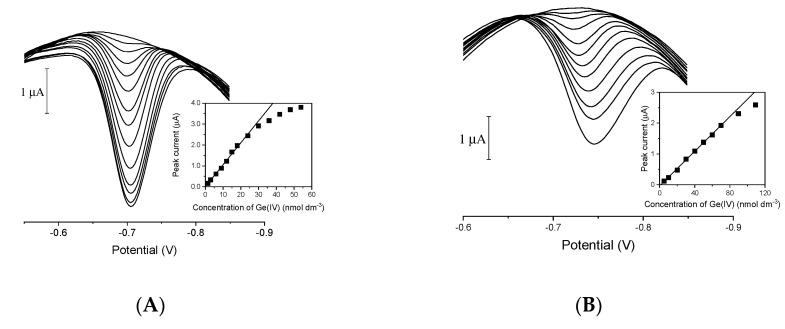

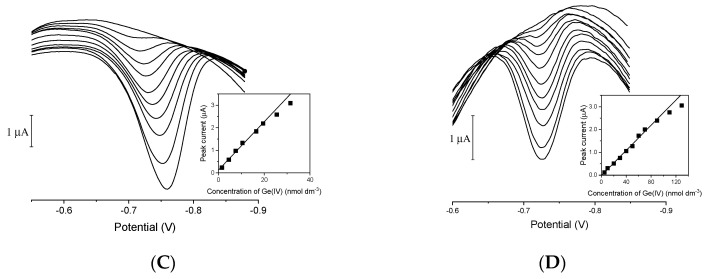
Voltammograms recorded using BiF/GC (**A**), BiF/SPE (**B**), BiF/SPE_meso_ (**C**), and BiF/SPE_or-meso_ (**D**) electrodes. The insets show corresponding calibration plots. Supporting electrolyte: 0.05 M acetate buffer, 1 mM of catechol, 1 mM of V(IV), and 1.5 mM of HEDTA. Instrumental parameters: accumulation time 30 s, accumulation potential −0.4 V. Other parameters as in Figure 1.

Finally, the dependence of the germanium peak current on germanium concentration was examined using BiFE/GC (Figure 7A), BiFE/SPE (Figure 7B), BiFE/SPE_meso_ (Figure 7C), and BiFE/SPE_or-meso_ (Figure 7D) in a solution containing 0.05 M acetate buffer, 1 mM of catechol, 1 mM of V(IV), and 1.5 mM of HEDTA, with 30 s of adsorptive accumulation at −0.4 V in a stirred solution. The calibration parameters presented in Table 1 show that procedures that employ BiFEs for the determination of Ge(IV) have the same advantages as those utilizing the HMDE electrode (namely high sensitivity and reproducibility of germanium(IV) CAdSV signals) [10].

Interference studies involving typical ions were described in our previous work [10]. In consideration of the metallic components commonly found in electronic waste, the inference studies were extended to include gold, silver, nickel, indium, and cobalt present in conductive paths, touchscreens, and batteries [32,33]. It was found that the presence of a 700-fold excess of Au(III), Ag(I), Co(II), Ni(II), Li(I), a 150-fold excess of Sn(IV), a 100-fold excess of copper and 30-fold excess of In(III) did not cause any significant change (>5%) in the Ge(IV) peak current. The addition of a 300-fold, 200-fold, or 100-fold excess of Sn(IV), Cu(II), and In(III), respectively, caused the germanium peak current to decay by 10% to 20%.

**Figure 8:**
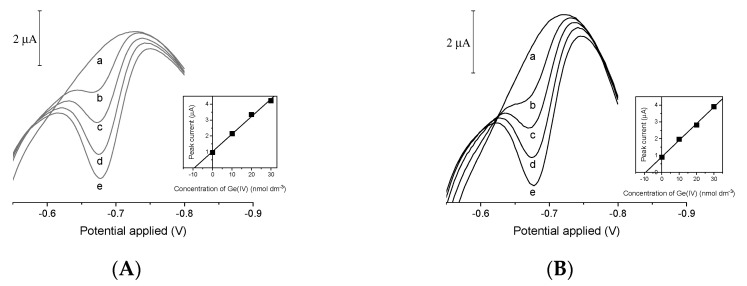
Voltammograms recorded by means of BiF/SPE (**A**) and BiF/SPE_meso_ (**B**) electrodes in seawater samples (a) and samples spiked with 10 nM of Ge(IV) (b). Voltammograms (c–e) were obtained after successive standard additions of 10 nM of Ge(IV). Insets show standard addition plots. Every calibration point represents the average value of three replicates. Supporting electrolyte: 0.05 M acetate buffer, 1 mM of catechol, 1 mM of V(IV), and 1.5 mM of HEDTA. Instrumental parameters: accumulation time 30 s, accumulation potential −0.4 V. Other parameters as in Figure 1.

The elaborated procedure was tested using real samples spiked with 10 nM of Ge(IV). As examples, Figure 8 shows the results obtained when BiFE/SPE (Figure 8A) and BiFE/SPE_meso_ (Figure 8B) were applied for Ge(IV) determination in seawater via the standard additions method. The overall recovery of germanium was 9.90 to 10.4 nM.

## 4. Conclusions

The application of the V(IV)-HEDTA complex to induce catalytic reactions involving Ge(IV) is advantageous for a number of reasons. It was shown that the V(IV)-HEDTA complex is very efficient at enhancing the germanium signals both when applying mercury electrodes [10,11,12] and bismuth film electrodes plated on a variety of supports. Satisfactory results were obtained in the case of both homogeneous supports (including glassy carbon and gold coated with a bismuth layer via PVD) and heterogeneous materials (carbon fibers encapsulated in a polymer, carbon, and mesoporous carbon screen-printed electrodes). On the other hand, the introduction of other oxidants such as bromate resulted in the complete loss of germanium signals, making the electrode unusable. Such strong oxidants attack both the deposited bismuth film and the SPE support.

The applied experiment design allowed the bismuth film deposition to be optimized, making the proposed Ge(IV) determination procedure even more effective. When the appropriate plating parameters were used, the geometry of Ge(IV) signals (height, width, and symmetry) was more favorable and, more importantly, the reproducibility of Ge(IV) signals greatly improved, reaching a level of 2%, which is very rare for SPEs.

The careful optimization of other accumulation parameters allowed a very sensitive procedure for the determination of germanium to be designed. All tested electrodes may be used to determine ultratrace levels of Ge(IV) with high sensitivity and low limits of detection (from 0.8 nM for BiFE/SPE_or-meso_ to 1.0 nM for BiFE/SPE and BiFE/GC) for an accumulation time as short as 30 s. The tests performed on natural samples showed that it was possible to perform a highly reliable determination of Ge(IV) in a real matrix using portable instrumentation. It can be concluded that the developed procedure can also be used for germanium mineral prospecting and exploration as well as for the screening of electronic waste leachates in the search for germanium-rich secondary raw materials that can be recycled with high profit margins.

## Figures and Tables

**Table 1:** Calibration parameters and corresponding standard errors of CAdSV procedure of Ge(IV) determination obtained by regression analysis.

Electrode Type	Calibration Formula *	Linear Range (nM)	R^2^	LOD (nM)
BiFE/GC	y = (0.100 ± 0.003)x + (0.05 ± 0.05)	1.5–24	0.9928	1.0
BiFE/SPE	y = (0.107 ± 0.004)x + (0.13 ± 0.05)	1.5–19.5	0.9945	1.0
BiFE/SPE_meso_	y = (0.0281 ± 0.0004)x + (−0.04 ± 0.02)	5.0–70	0.9985	1.2
BiFE/SPE_or-meso_	y = (0.0276 ± 0.0009)x + (−0.02 ± 0.04)	5.0–90	0.9931	0.8

* y and x denote peak current (μA) and Ge(IV) concentration (nM); to construct the calibration curve, three replicates for each concentration were considered.

## Data Availability

The data presented in this study are available on request from the corresponding author.

## Supplementary Materials

The following are available online at https://www.mdpi.com/article/10.3390/membranes11070524/s1: Scheme S1. Laboratory made multifibre electrode; Table S1. Design matrix for response surface quadratic model; Table S2. Interpretation of the germanium peaks shown in Figure 5.
