# Supplementary material for: Catalytic Adsorptive Stripping Voltammetric Determination of Germanium Employing the Oxidizing Properties of V(IV)-HEDTA Complex and Bismuth-Modified Carbon-Based Electrodes
Source: Membranes (Basel). 2021 Jul 13;11(7):524. doi: 10.3390/membranes11070524 (PMC8308015; doi:10.3390/membranes11070524)
Supplement: Supplementary file 1 [file membranes-11-00524-s001.zip › membranes-1285275-supplementary.pdf]

Supplementary material

Catalytic adsorptive stripping voltammetric determination of germanium employing the oxidizing properties of V(IV)-HEDTA complex and bismuth-modified carbon-based electrodes

Agnieszka Królicka*, Jerzy Zarębski and Andrzej Bobrowski

Department of Building Materials Technology, Faculty of Materials Science and Ceramics, AGH University of Science and Technology, Mickiewicza 30, 30-059 Krakow, Poland; krolicka@agh.edu.pl

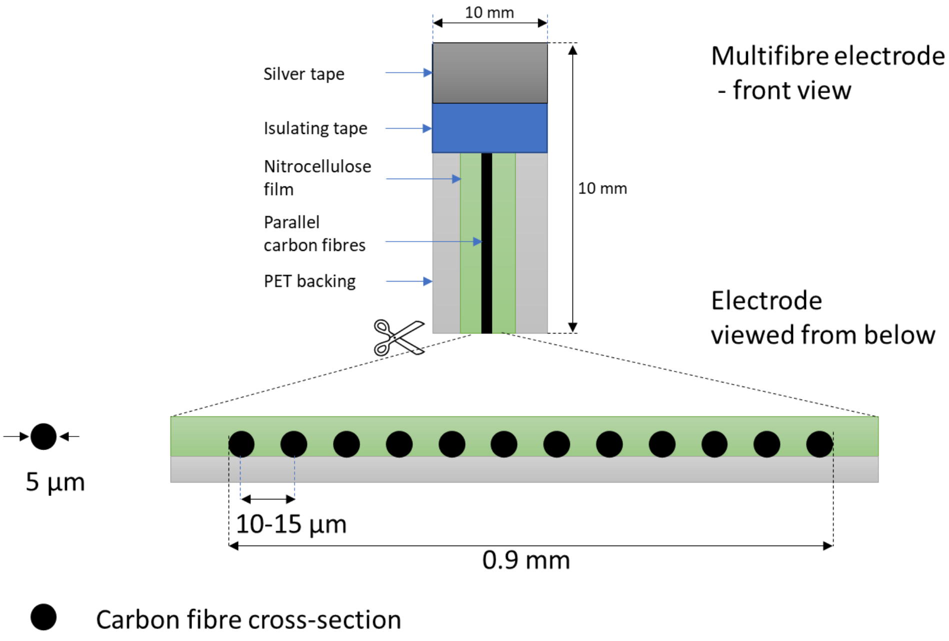

Scheme S1. Laboratory made multifibre electrode.

Table S1. Design matrix for response surface quadratic model.

No	x_1	x_2	x_3	E_{plat} (V)	Q* (mC)	c (mM)	t_1	t_2	Peak current (µA) (t_1)	Peak current (µA) (t_2)	$w_{1/2}$ (V) (t_1)	$w_{1/2}$ (V) (t_2)
1	-1	-1	-1	-0.9	10	13	10	14	3.6	3.8	0.067	0.065
2	1	-1	-1	-1.5	10	13	11	4	4.7	4.1	0.068	0.062
3	-1	1	-1	-0.9	50	13	14	6	5.6	4.7	0.066	0.058
4	1	1	-1	-1.5	50	13	7	5	4.1	4.7	0.065	0.066
5	-1	-1	1	-0.9	10	43	2	9	3.8	2.7	0.068	0.077
6	1	-1	1	-1.5	10	43	15	11	5.0	5.0	0.064	0.064
7	-1	1	1	-0.9	50	43	8	13	5.9	6.0	0.062	0.065
8	1	1	1	-1.5	50	43	3	8	4.9	5.8	0.056	0.061
9	0	0	0	-1.2	30	28	12	7	4.78	5.0	0.065	0.065
10	-1	0	0	-0.9	30	28	4	3	3.80	4.4	0.070	0.069
11	1	0	0	-1.5	30	28	13	10	4.76	5.1	0.062	0.065
12	0	-1	0	-1.2	10	28	6	2	1.53	4.0	0.062	0.066
13	0	1	0	-1.2	50	28	9	1	5.88	5.3	0.062	0.061
14	0	0	-1	-1.2	30	13	5	15	4.38	3.3	0.063	0.063
15	0	0	1	-1.2	30	43	1	12	3.02	5.3	0.060	0.069

*value for electrodes with a surface area of 7.07 square millimeters (GC disc with a diameter of 3 mm)

$w_{1/2}$ – the half-width peak potential

t_1 and t_2 columns represent the random numbers indicating the order of experiments in trial 1 and trial 2. Every trial comprised 15 combinations of x_1, x_2, and x_3 factors

Table S2. Interpretation of the germanium peaks shown in Figure 5.

Panel	Electrode type	Shape	Area (mm^2)	$C_{Ge(IV)}$ (nM)	$E_{p\ (a)}$ (V)	$E_{p\ (b)}$ (V)	$E_{p\ (a)}$ - $E_{p(b)}$ (V)	$I_{p(a)}$ (µA)	$I_{p(b)}$ (µA)	$I_{p(a)}$ - $I_{p(b)}$ (µA)
A	BiFE/GC	Circle	7.07	30	-0.705	-0.705	0	2.54	2.63	0.08
B	BiFE/SPE	Circle	12.57	30	-0.739	-0.76	-0.021	2.00	2.13	0.13
C	BiFE/SPE_{meso}	Circle	12.57	30	-0.739	-0.751	-0.012	0.91	0.86	-0.06
D	BiFE/$SPE_{or\text{-}meso}$	Circle	12.57	30	-0.7	-0.705	-0.005	0.87	0.88	0.01
E	BiFE/SPE_g	Circle	12.57	30	-0.726	-0.763	-0.037	8.19	6.67	-1.53
F	SPE/rGO	Circle	12.57	30	-0.705	-0.72	-0.015	2.08	1.17	-0.92
G	BiFE/F	Array of circles	< 0.005	300	-0.766	-0.77	-0.004	0.005	0.005	0.00
H	Bi_{sp}	Square	80.00	300	-0.748	-0.775	-0.027	4.58	6.04	1.46
